# Supplementary material for: Integrated mechanical computing for autonomous soft machines
Source: Nat Commun. 2024 Apr 4;15:2933. doi: 10.1038/s41467-024-47201-y (PMC10995184; doi:10.1038/s41467-024-47201-y)
Supplement: Supplementary file 1 — Supplementary Information [file 41467_2024_47201_MOESM1_ESM.pdf]

Supplementary Information for

Integrated Mechanical Computing for Autonomous Soft Machines

Junghwan Byun,[1,2]† Aniket Pal,[1,3]† Jongkuk Ko,[1,4] Metin Sitti[1,5,6]*

[1]Physical Intelligence Department, Max Planck Institute for Intelligent Systems, 70569 Stuttgart, Germany.

[2]Soft Hybrid Materials Research Center, Korea Institute of Science and Technology, 02792 Seoul, Republic of Korea.

[3]Institute of Applied Mechanics, University of Stuttgart, 70569 Stuttgart, Germany.

[4]Department of Chemical and Biological Engineering, Gachon University, Gyeonggi-do 13120, Republic of Korea.

[5]Institute for Biomedical Engineering, ETH Zürich, 8092 Zürich, Switzerland.

[6]School of Medicine and College of Engineering, Koç University, 34450 Istanbul, Turkey.

†These authors contributed equally to this work.

*Correspondence to: sitti@is.mpg.de

The PDF file includes:

Supplementary Figs. 1-19

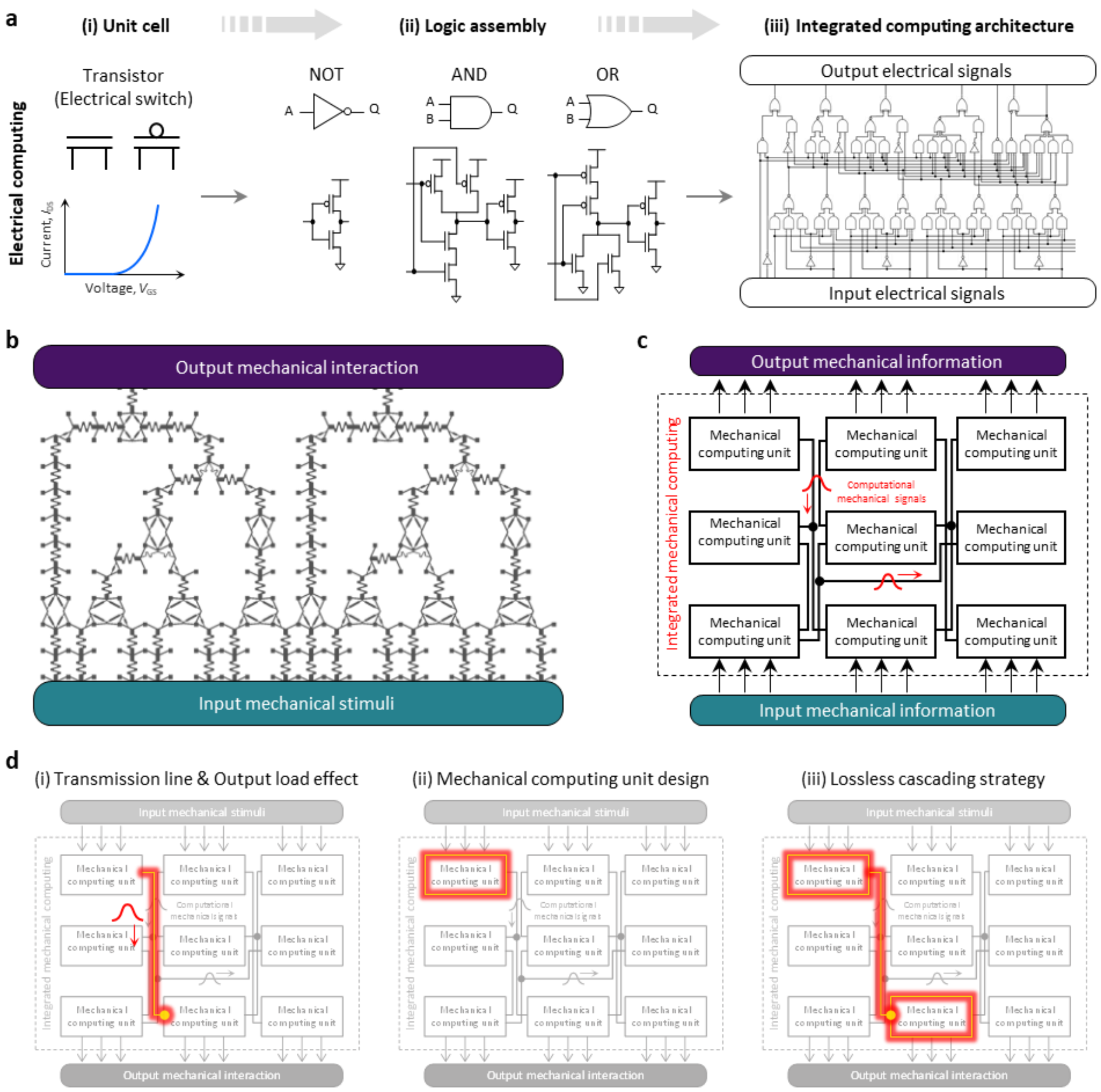

Supplementary Fig. 1 | Inspiration and design requirements for integrated mechanical computing. a, Unit cells, their topological assembly for computational logics, and integrated system architectures for conventional electrical computing **b**,**c**, A representative mechanical analogue of integrated computing (**b**) and its schematic diagram (**c**). As in its electrical counterpart, the integrated system architecture for mechanical computing needs to combine networked mechanical computing units that map environmental input mechanical stimuli to the output mechanical interactions. **d**, Key design requirements for integrated mechanical computing are threefold: (i) The optimal mechanical transmission line needs to be developed for stable propagation of mechanical information. The engineering of its input/output characteristics is also critical to deal with output load effects induced by the effective mechanical impedance of a single computing unit or its cascaded form. (ii) Rational designs of mechanical computing units ("mechanologics"), which allow for not only compact, deterministic computational functions, but also accessible modular connectivity, need to be developed. (iii) A systematic approach for achieving lossless computation through multiple cascaded mechanologics needs to be established.

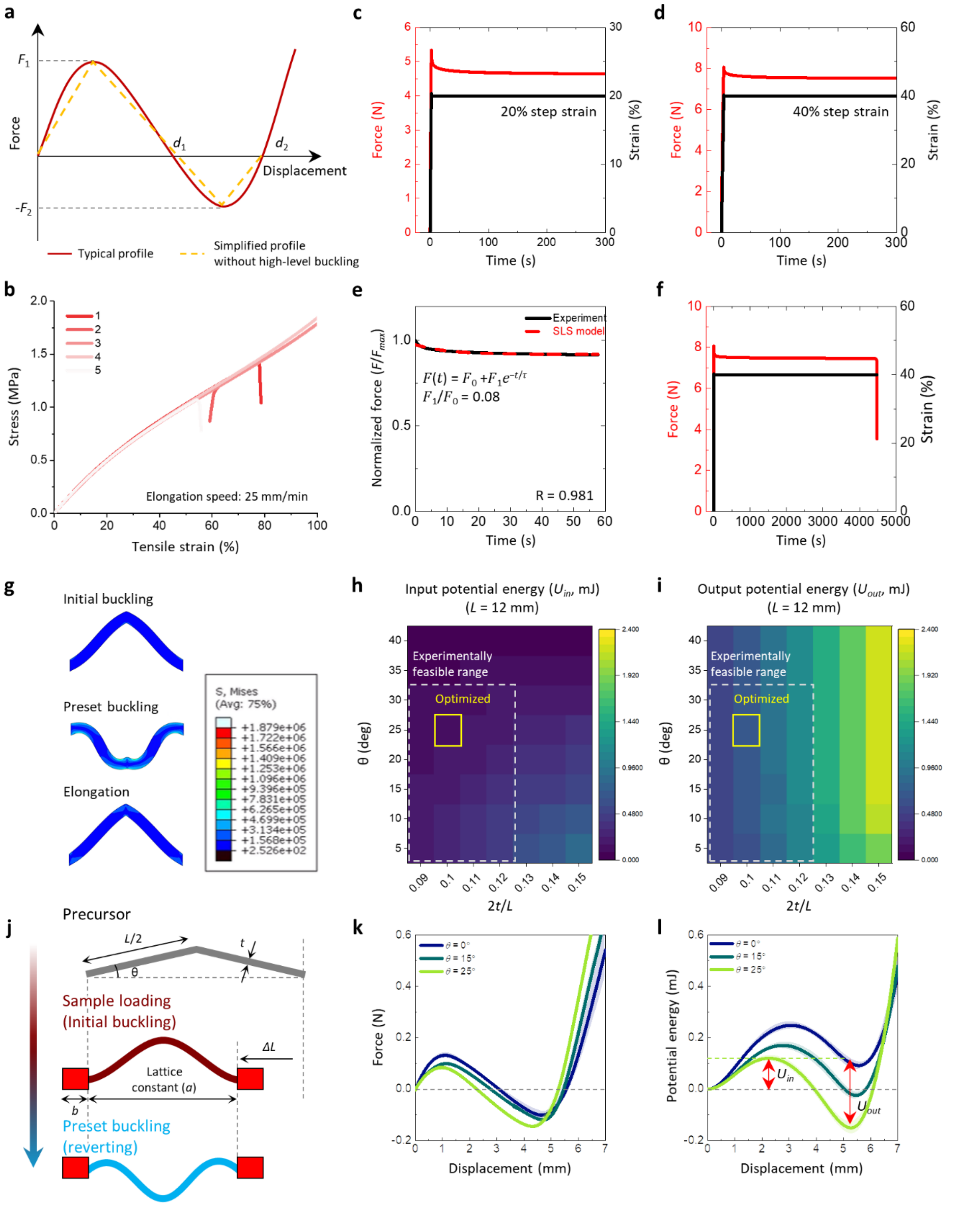

Supplementary Fig. 2 | Material, design optimization, and characterization of a soft bistable unit element. a, A typical force-displacement profiles of a buckled bistable beam with (red) and without (yellow) consideration of high-level buckling modes[54-56]. **b**, Elongation test (speed: 25 mm min^{-1}) results of the Elastic 50A material to be used for numerical studies. The results of the five different tests suggest the Young's modulus of ~2.04 MPa with elongation at break of ~60%. **c**,**d**, Stress relaxation experimental results with dogbone samples fabricated with the Elastic 50A material, where step strains of 20% (**c**) and 40% (**d**) were applied and maintained to the sample and the force was measured for 300 s. **e**, Fitted Maxwell form of the

standard linear solid (SLS) model of linear viscoelasticity (correlation coefficient R = 0.981). **f**, Long-term stress relaxation behavior at 40% strain. **g**, Sequential steps of numerical studies for a single bistable element. The simulation starts with the lateral compression step for initial buckling, exerts a prescribed vertical displacement to reach the preset state, and finally applies a vertical load at constant velocity to revert the beam (see Materials and Methods for details). **h**,**i**, Input (**h**) and output (**i**) potential energies of a soft bistable element as a result of numerical parametric studies. **j**, Schematic illustration of the experimental process for loading and characterizing a soft bistable beam whose geometry is defined by a parameter set of (a, L, t, θ). Red squares indicate the rigid lattice frame. **k**,**l**, Experimental characterization of typical force (**k**) and energy (**l**) profiles of soft bistable beams defined by a parameter set of (a, L, t) = (9 mm, 12 mm, 0.6 mm). Error bounds indicate SD. Given the small U_{in} and large U_{out}, our design process was based on the beam with θ=25°.

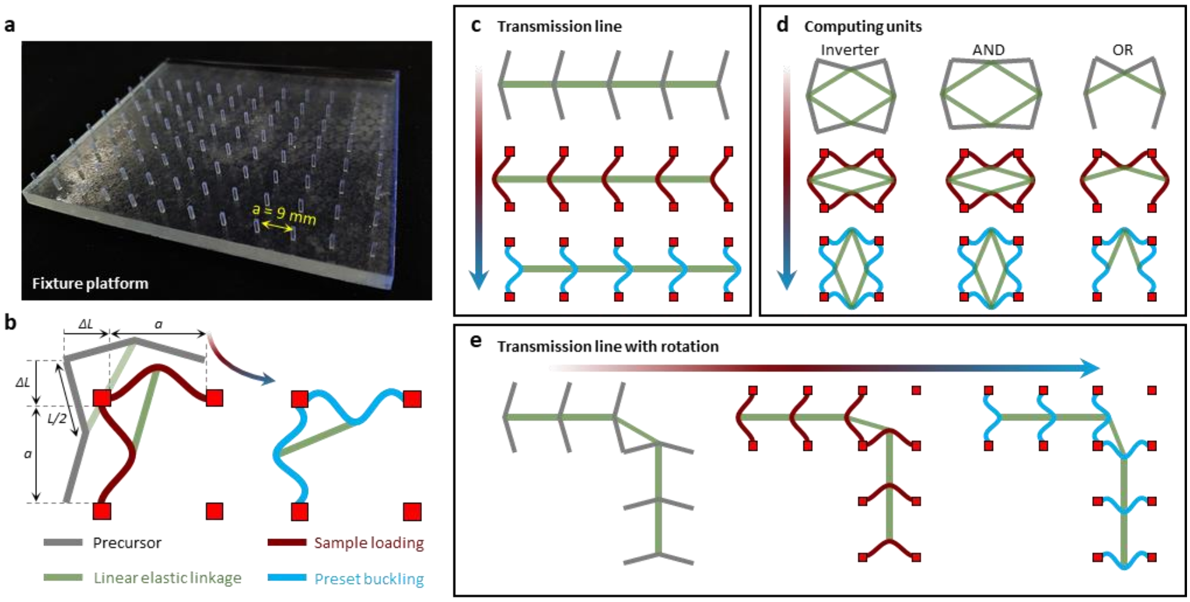

Supplementary Fig. 3 | Strategic topological assembly for functional components in mechanical computing systems. a, Photograph of a 3D printed lattice frame with a lattice constant (a) of 9 mm. **b**, Schematic illustration of the placement process of networked bistable elements onto the rigid lattice frame. **c**-**e**, Topological assemblies and placement processes for a mechanical transmission line (**c**), mechanologics (**d**), and a transmission line with a redirector unit (**e**).

a

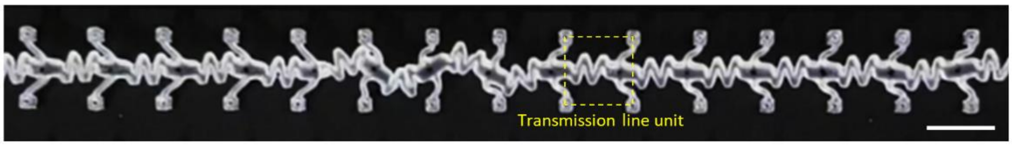

b

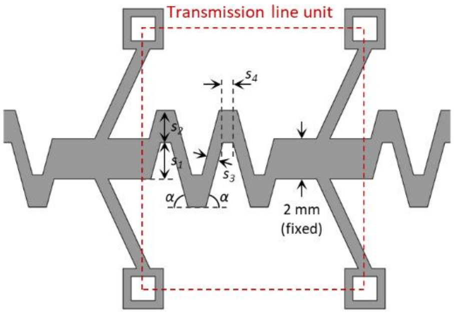

c

	α (°)	S_1(mm)	S_2 (mm)	S_3 (mm)	S_4 (mm)
**V0**	75	2.4	0.6	0.6	0
**V1**	75	2	1.6	0.6	0
**V2**	75	2	1.6	0.8	0
**V3**	75	1.6	1.6	0.6	0
**V4**	75	1.6	1.6	0.8	0
**V5**	75	1.2	1.6	0.6	0
**V6**	75	1.2	1.6	0.6	0.5
**V6-1**	75	1.5	1.6	0.6	0.5
**V6-2**	75	1.8	1.6	0.6	0.5

d e

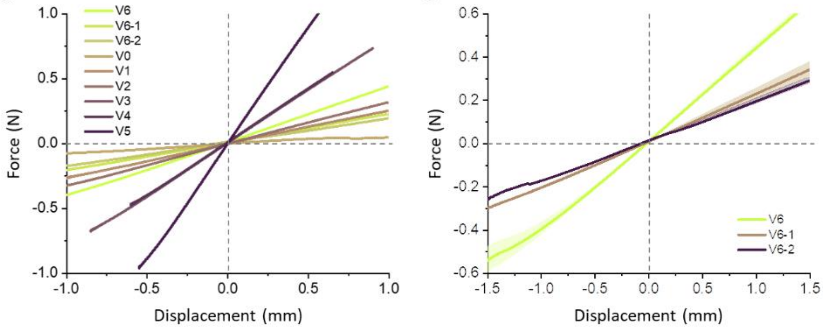

f

	k (N/m)
**V0**	80
**V1**	270
**V2**	330
**V3**	830
**V4**	820
**V5**	1780
**V6**	400
**V6-1**	210
**V6-2**	180

Supplementary Fig. 4 | Design of mechanical transmission lines. a, Photograph of a mechanical transmission line, reproduced from Fig. 2a. Scale bar, 1 cm. **b**, Schematic drawing of a transmission line unit consisting of a bistable beam and a spring-like linear elastic linkage structure. **c**, An experimentally explored design space for the spring structure, with parameters defined in (**b**). **d**-**f**, Experimentally obtained force-displacement curves for each spring design (**d**), curves for the designs of interest (**e**), and the calculated spring stiffnesses (**f**).

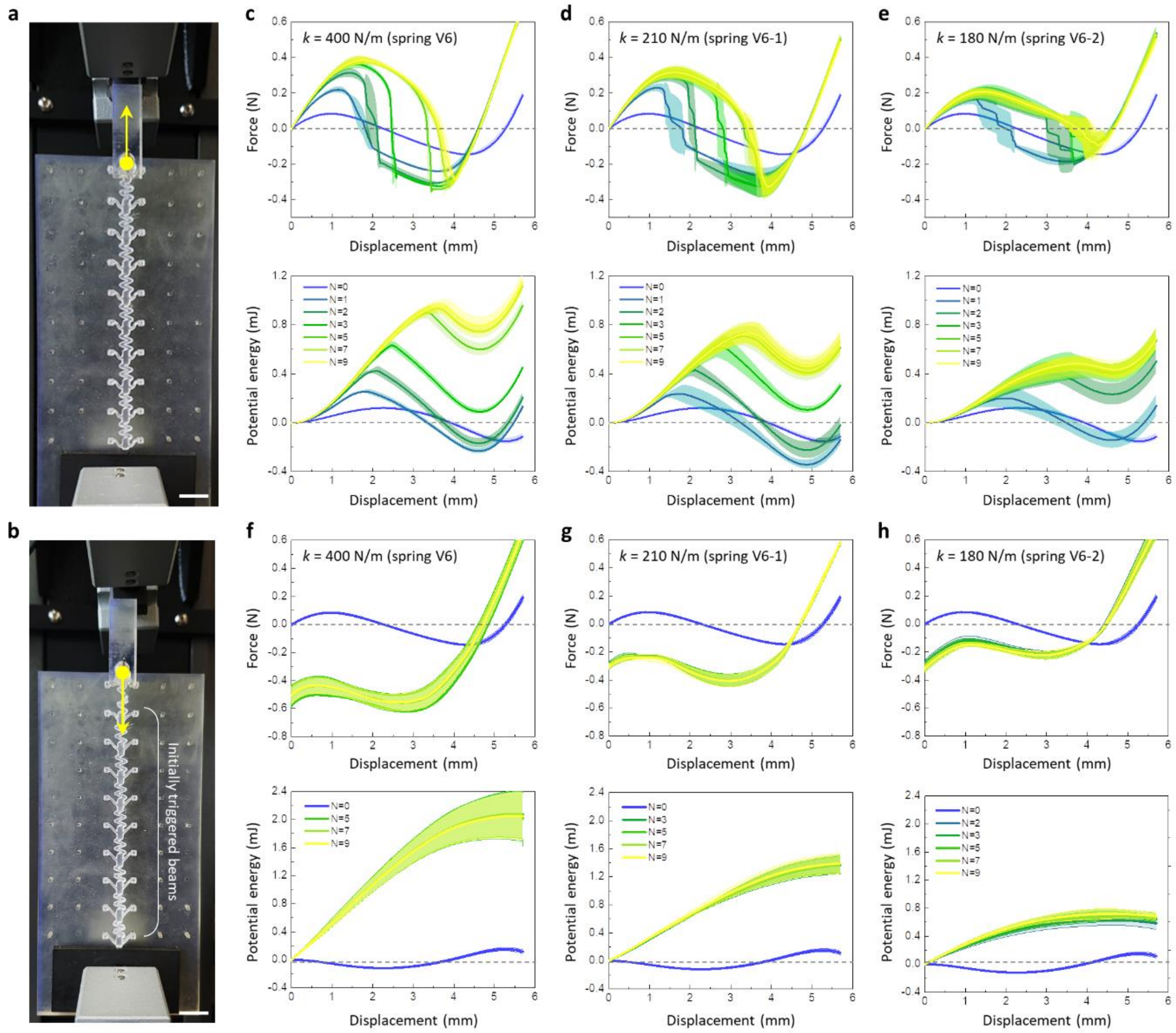

Supplementary Fig. 5 | Design optimization and experimental characterization of mechanical transmission lines. a,**b**, Photograph of the experimental setup for measuring the input (**a**) and output (**b**) characteristics of mechanical transmission lines. Scale bars, 1 cm. For the input measurement, *N*+1 bistable beams (i.e., *N* springs) were preset in a downward direction and then constantly pulled up at a rate of 4 mm min^{-1} (A yellow arrow indicates the direction of loading). For the output measurement, *N*+1 bistable beams were upwardly preset and then the bottom *N* beams were initially triggered to apply the output energy of the transmission line ($U_{out}^{T(N)}$) to the one fixed by the load cell. Finally, the beam was constantly pushed down at a rate of 4 mm min^{-1}. Assuming that the transmission line possesses similar responses to the pushing and pulling forces, subtraction between the measured force-displacement profile and the characteristic profile of a unit beam provided net output characteristics. **c**-**e**, Input force (top) and potential energy (bottom) characteristics of mechanical transmission lines for different *N* (number of springs) with spring stiffness *k* of 400 N m^{-1} (**c**), 210 N m^{-1} (**d**), 180 N m^{-1} (**e**). **f**-**h**, Output force (top) and potential energy (bottom) characteristics of mechanical transmission lines for different *N* with *k* of 400 N m^{-1} (**f**), 210 N m^{-1} (**g**), 180 N m^{-1} (**h**).

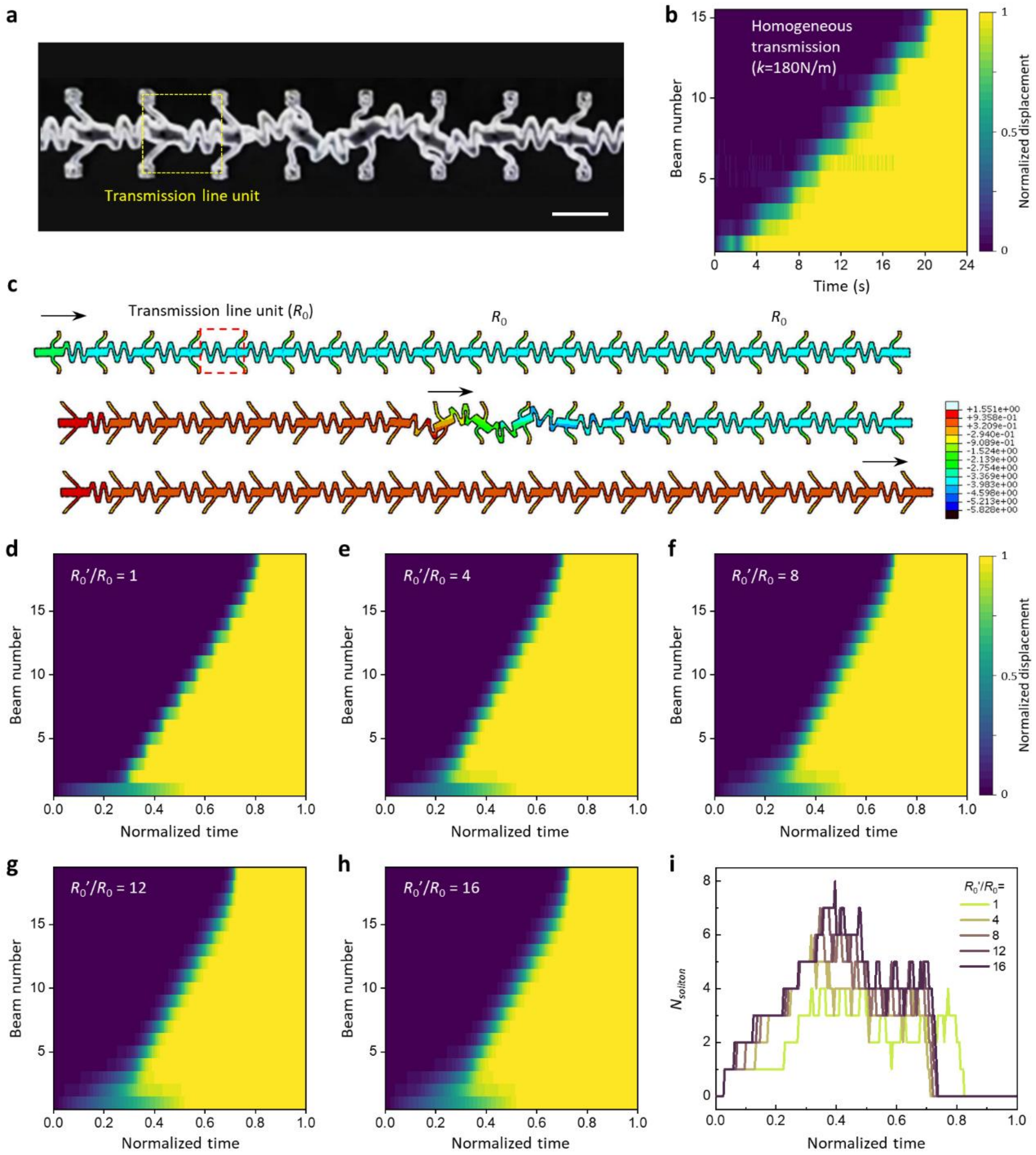

Supplementary Fig. 6 | Experimental and numerical studies of homogeneous propagation with the investigation of $N_{soliton}$. a, Typical behavior of mechanical signals propagating through a homogeneous transmission line (Supplementary Movie 1), reproduced from Fig. 2a. Scale bar, 1 cm. **b**, Time-evolving normalized displacement data for the homogeneous propagation shown in (**a**). **c**, Transition wave-based homogeneous propagation through the mechanical transmission line implemented in finite element analysis. The system consists of 19 bistable beams, defined by an optimal parameter set of (a, L, t, θ) = (9 mm, 12 mm, 0.6 mm, 25°) (see Supplementary Fig. 2 and Methods), and 18 springs of variable spring stiffness k. **d-h**, Evolution of the normalized displacement of each bistable unit in homogeneous propagation with the unit impedance of R_0' equal to R_0 (**d**), $4R_0$ (**e**), $8R_0$ (**f**), $12R_0$ (**g**), and $16R_0$ (**h**). **i**, Evolution of $N_{soliton}$ as a function of normalized time for the propagations shown in (**d-h**).

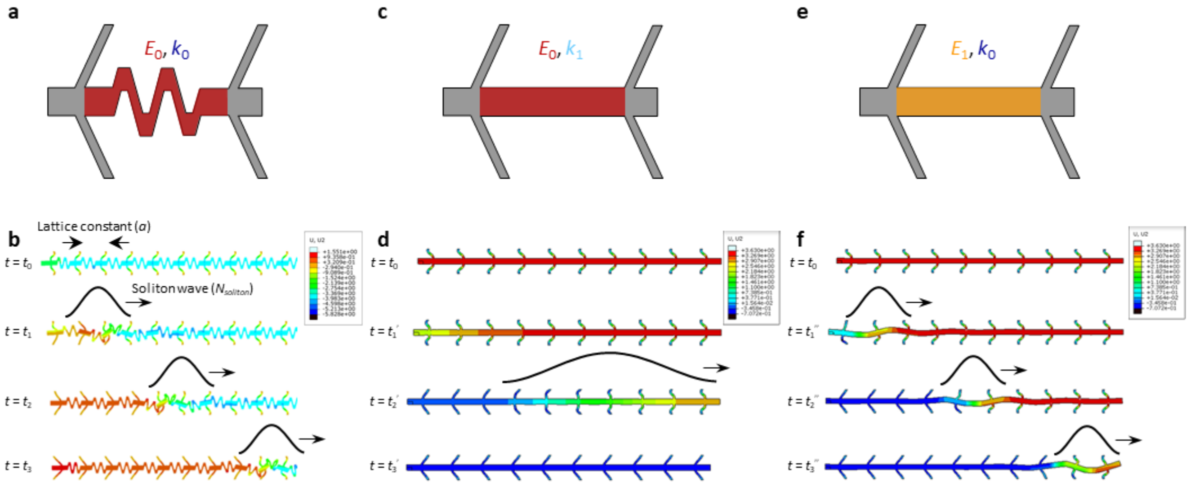

Supplementary Fig. 7 | Numerical studies of solitary wave motion within mechanical transmission lines. a, Schematic illustration of the transmission line unit with a spring-like asymmetric serpentine linkage having an elastic modulus of E_0 (=2.04 MPa) and a spring stiffness of k_0 (=180 N m^{-1}) (see the design V6-2 shown in Supplementary Fig. 4b,c). **b**, Simulation result of time-evolving solitary wave propagation through the transmission line with unit element in A, where the soliton width ($aN_{soliton}$) is estimated as ~2.5*a*. **c**,**e**, Schematic illustration of transmission line units with spring-like symmetric straight linkages having an elastic modulus and a spring stiffness (E, k) of (E_0, k_1) (**c**) and (E_1, k_0) (**e**), respectively. **d**,**f**, Simulation results of time-evolving solitary wave propagation through the transmission lines with unit elements in C and E, respectively, where the soliton width is estimated as ~7*a* (**d**) and ~2.5*a* (**f**), respectively.

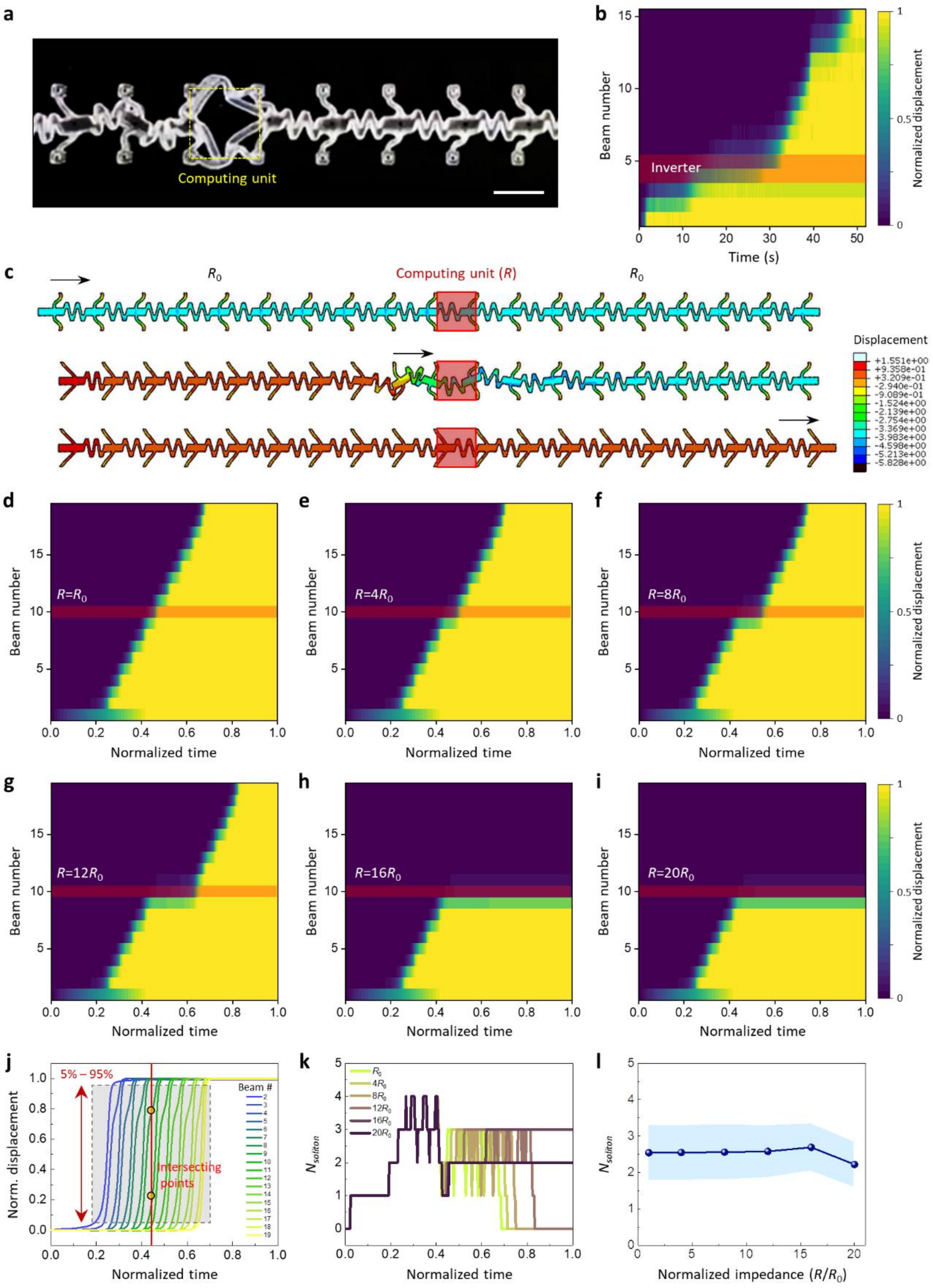

Supplementary Fig. 8 | Experimental and numerical studies of computational propagation through a computing unit. **a**, Typical behavior of mechanical signals propagating through a mechanical computing unit with structural (Supplementary Movie 1), reproduced from Fig. 2f. Scale bar, 1 cm. **b**, Time-evolving normalized displacement data for the computational propagation shown in (**a**). **c**, Solitary wave-based computational propagation implemented in finite element analysis (Supplementary Movie 2). The system consists of 19

bistable beams, defined by an optimal parameter set of (a, L, t, θ) = (9 mm, 12 mm, 0.6 mm, 25°), and 18 springs of k_0=180 N m^{-1}. Note that the mechanical computing unit is abstracted as the 10th spring geometry with variable mechanical impedances (R). **d**-**i**, Evolution of the normalized displacement of each bistable unit in computational propagation through a computing unit with an effective impedance (R) equal to R_0 (**d**), 4R_0 (**e**), 8R_0 (**f**), 12R_0 (**g**), 16R_0 (**h**), and 20R_0 (**i**). We see that, with increase in R, the propagation delay (computing process) becomes larger, and the propagation is eventually inhibited when R≥16R_0. **j**, The process of estimating $N_{soliton}$. See Methods for details. **k**, Evolution of $N_{soliton}$ as a function of normalized time for the computational propagations shown in (**d**-**i**). **l**, The numerically estimated $N_{soliton}$ values for computational propagations through a computing unit with various R. The error bound indicates SD. Note that $N_{soliton}$ is kept invariant regardless of the impedance of the computing unit, that is, irrespective of the type and geometry of mechanologics.

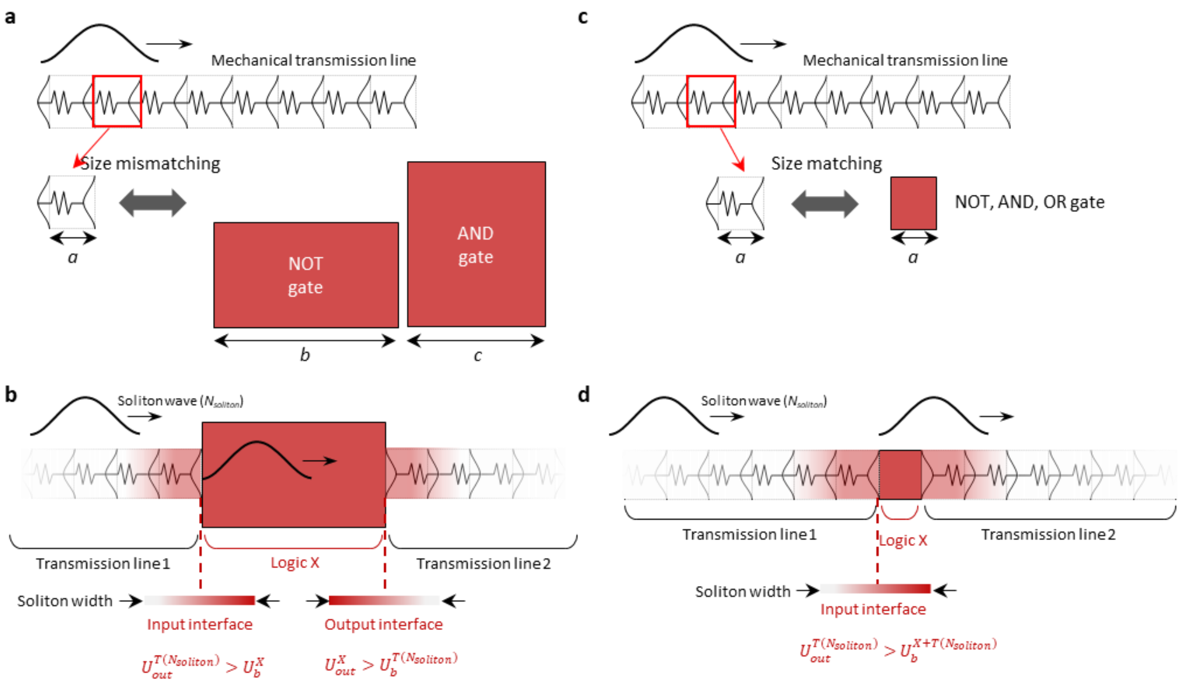

Supplementary Fig. 9 | Size matching between mechanologics and the transmission line unit. a, The dimension of existing mechanologics is not compact enough to match those of the transmission line unit (*b*, *c*>>*a*). **b**, The size mismatching in computational propagations not only makes the whole system bulky, but also complicates the potential energy analysis for successful propagation. **c**, The proposed mechanologic design, which can be defined within a unit lattice (length, *a*) achieves a size matching with the transmission line unit. **d**, The resultant computing platform consisting of mechanologics and mechanical transmission lines allows for i) superior system compactness, ii) seamless computation, iii) ease of systematic investigation of computational propagations due to the unified unit length (*a*) of transmission and computing events, and iv) simplification of energy barrier problem of mechanologics with transmission lines connected behind them.

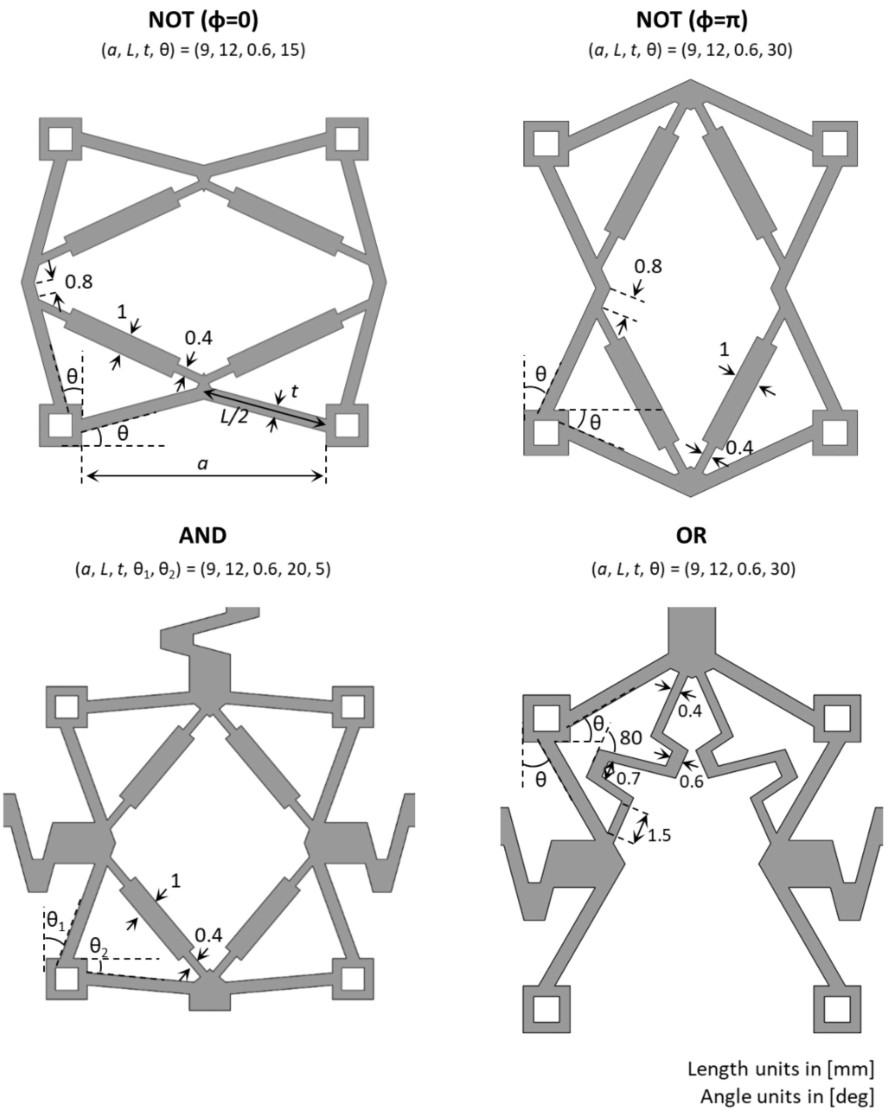

Supplementary Fig. 10 | Design specifications of mechanologics (NOT, AND, and OR gates).

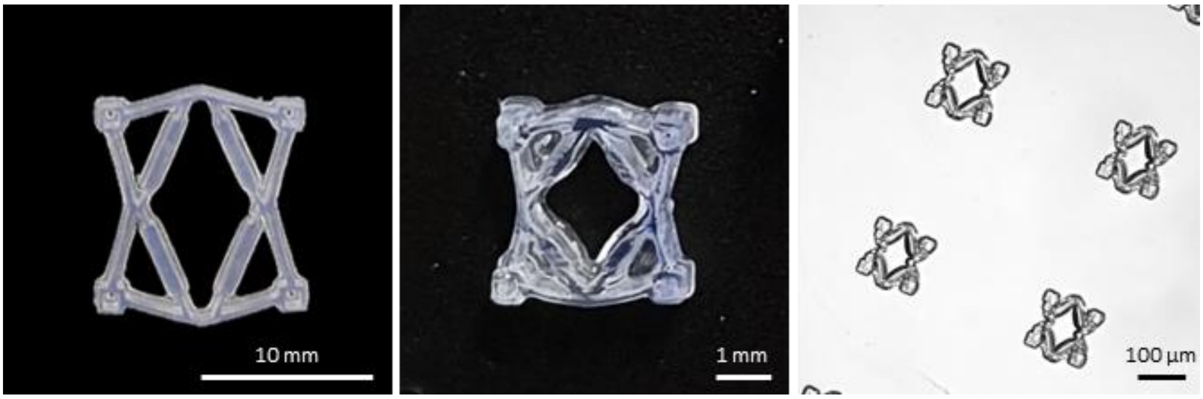

Supplementary Fig. 11 | 3D printed mechanologics in multiple scales. The centimeter-scale (left) and millimeter-scale (middle) mechanologics were fabricated by stereolithography 3D printing (Form 3B, Formlabs), while the micrometer-scale (right) mechanologics were fabricated by two-photon lithography 3D printing (Photonic Professional GT, Nanoscribe GmbH).

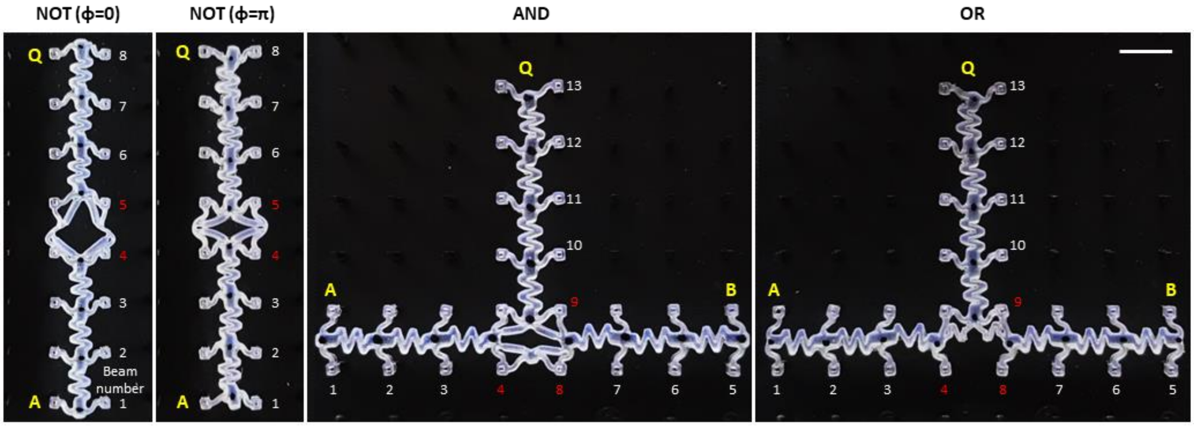

Supplementary Fig. 12 | Beam configurations for mechanologic analyses (Fig. 3e). Scale bar, 1cm.

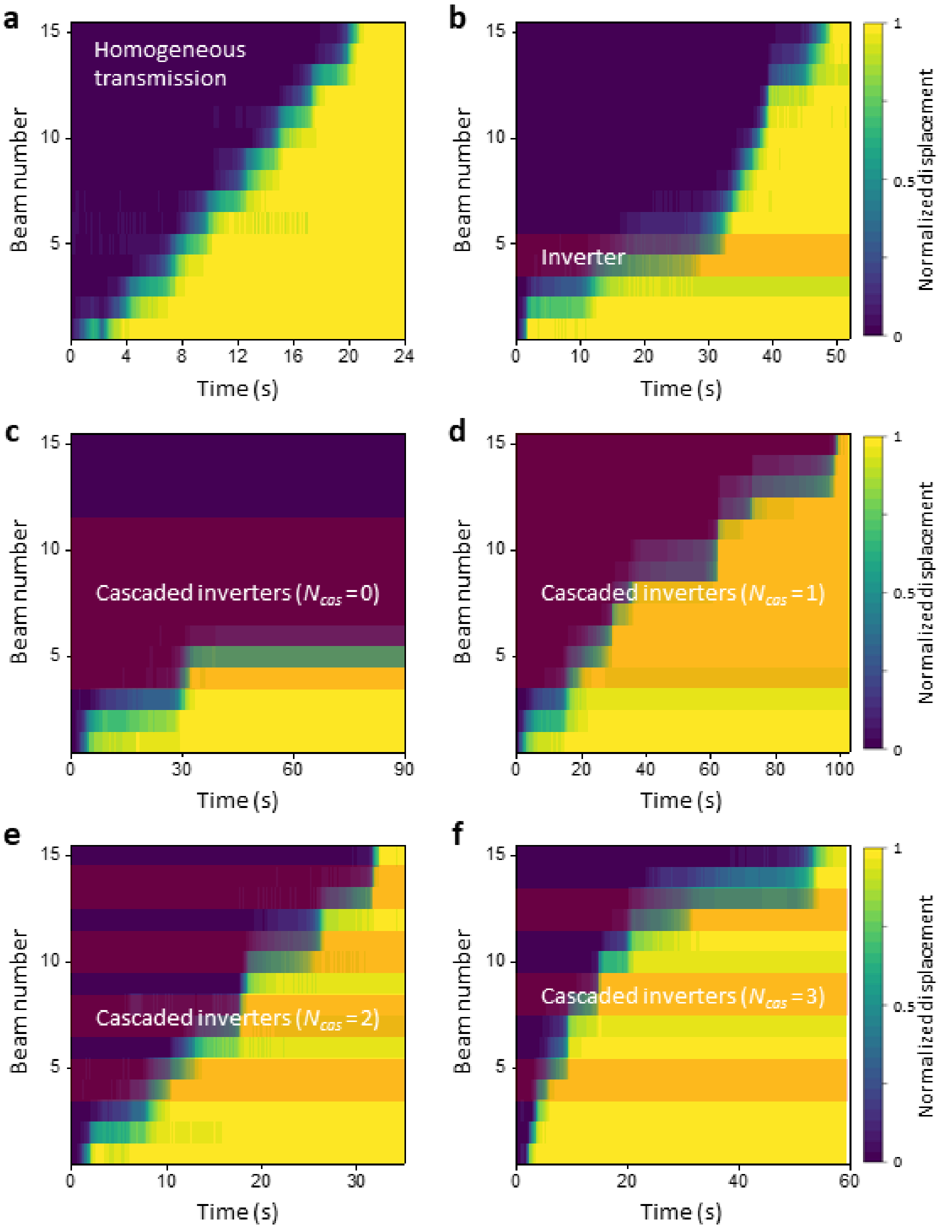

Supplementary Fig. 13 | Experimental characterization of cascaded computation shown in Fig. 4d and Supplementary Movie **7.** Evolution of the normalized displacement of each bistable unit in homogeneous propagation (**a**), in computational propagation through a single NOT gate (**b**), and in cascaded computing via networked NOT gates with N_{cas}=0 (**c**), N_{cas}=1 (**d**), N_{cas}=2 (**e**), N_{cas}=3 (**f**).

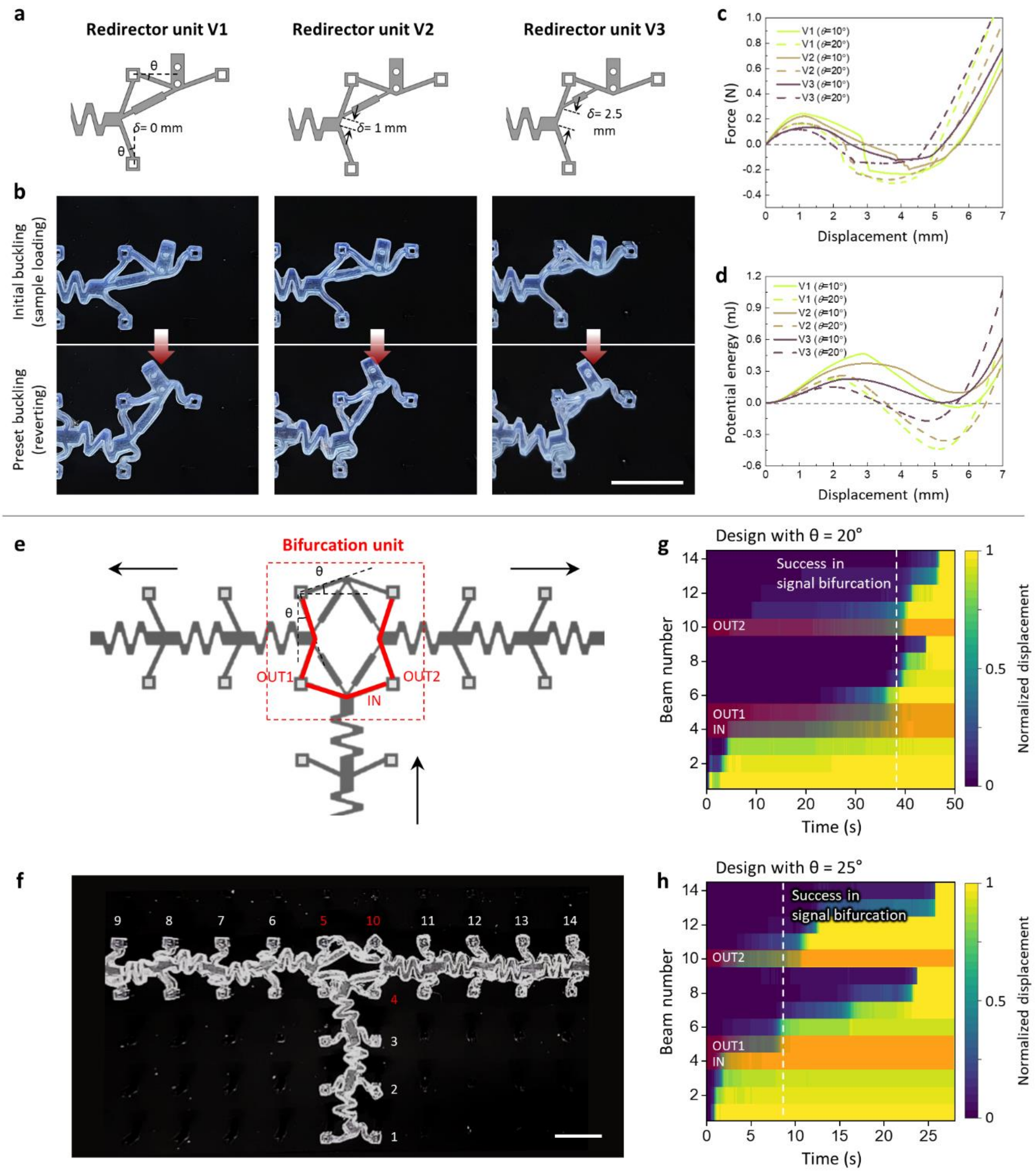

Extended Data Fig. 14 | Design optimization and experimental characterization of redirector and bifurcation units. a, Schematic drawing of redirector units in different design versions. For the redirector units, two bistable elements with the same geometry are arranged perpendicular to each other. **b**, Experimental demonstration of the designs shown in (**a**). Scale bar, 1 cm. **c**,**d**, Input/output characteristics of the redirector units. Given the data, the redirector unit V3 with θ=20° was chosen as an optimal design. The error bound indicates SD. **e**, Design specification of a bifurcation unit connected with mechanical transmission lines. **f**, Beam configurations for bifurcation analyses. Scale bar, 1 cm. **g**,**h**, Experimental characterization of signal bifurcation through the design with θ=20° (**g**) and 25° (**h**). Evolution of the normalized displacement of each bistable unit represents the difference in propagation behavior, such as speed, depending on the key geometric parameter, θ.

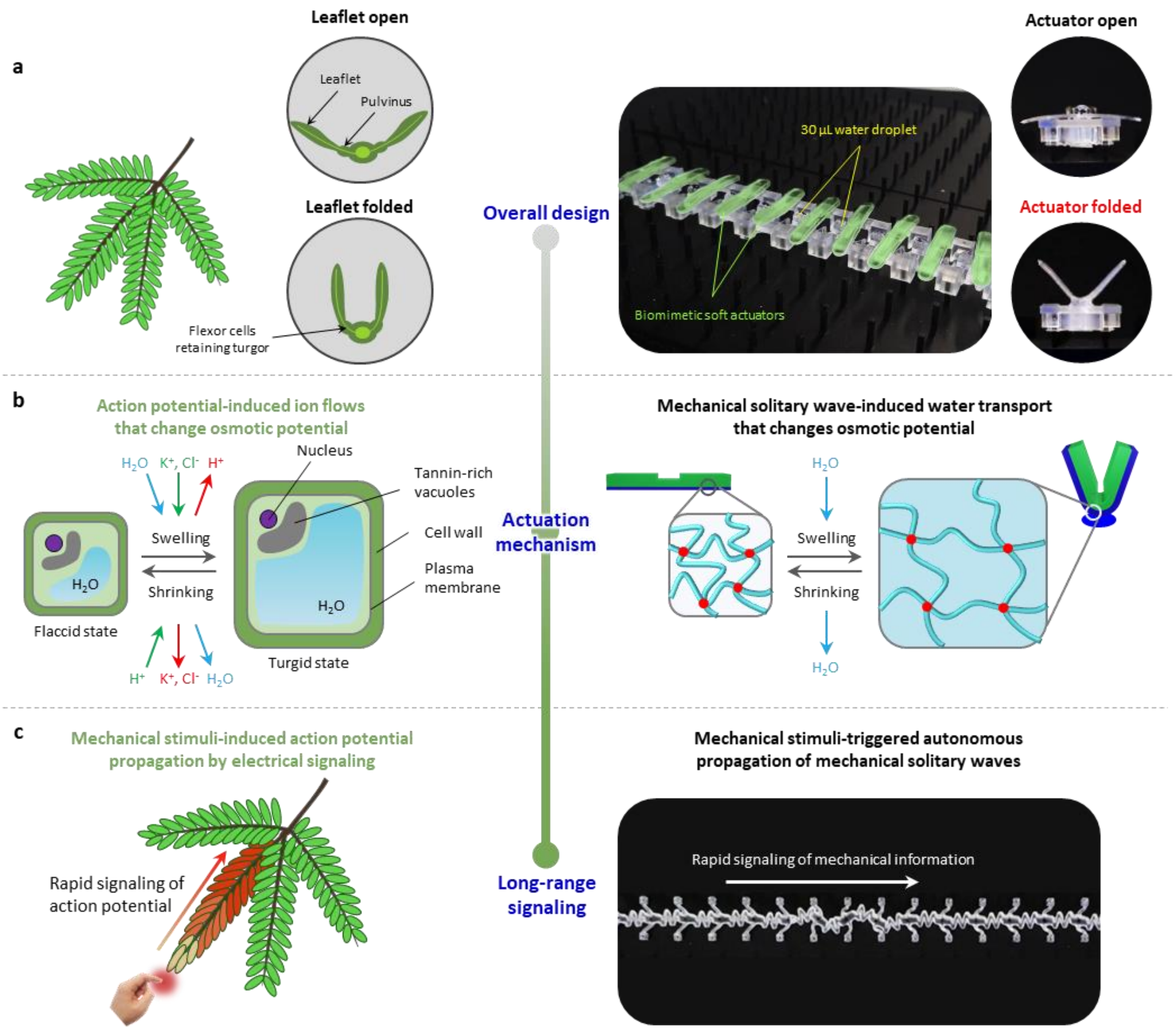

Supplementary Fig. 15 | Design concept of autonomous soft machines inspired by *M. pudica*. a, An overall design scheme for Mimosa-inspired soft machines that mimic the following behaviors and functions: (i) Leaflet folding in response to mechanical stimuli, (ii) swelling-induced actuation mechanism, and (iii) consecutive propagation of mechanical shape morphing from the site of stimulation. **b**, In *M. pudica*, a mechanical stimulus triggers an action potential that is transmitted to the pulvini, causing ion flows and the resulting changes in osmotic potentials. The outcome is the osmosis-driven water flows out of the extensor cells and into the flexor cells, generating leaflet folding. Inspired by this actuation mechanism, our design concept allows that a mechanical stimulus triggers a mechanical solitary wave that transports water droplets of sufficient volume; upon the uptake of water droplets being transported, the soft actuator made of rapidly swellable hydrogels experiences osmotic gradients, leading to the folding of the actuator. **c**, In *M. pudica*, an action potential can be autonomously propagated throughout the entire branch, and this long-range signaling delivers consecutive propagation of leaflet shape morphing. Inspired by this signaling behavior, our design concept combines the mechanical transmission line with soft hydrogel actuators.

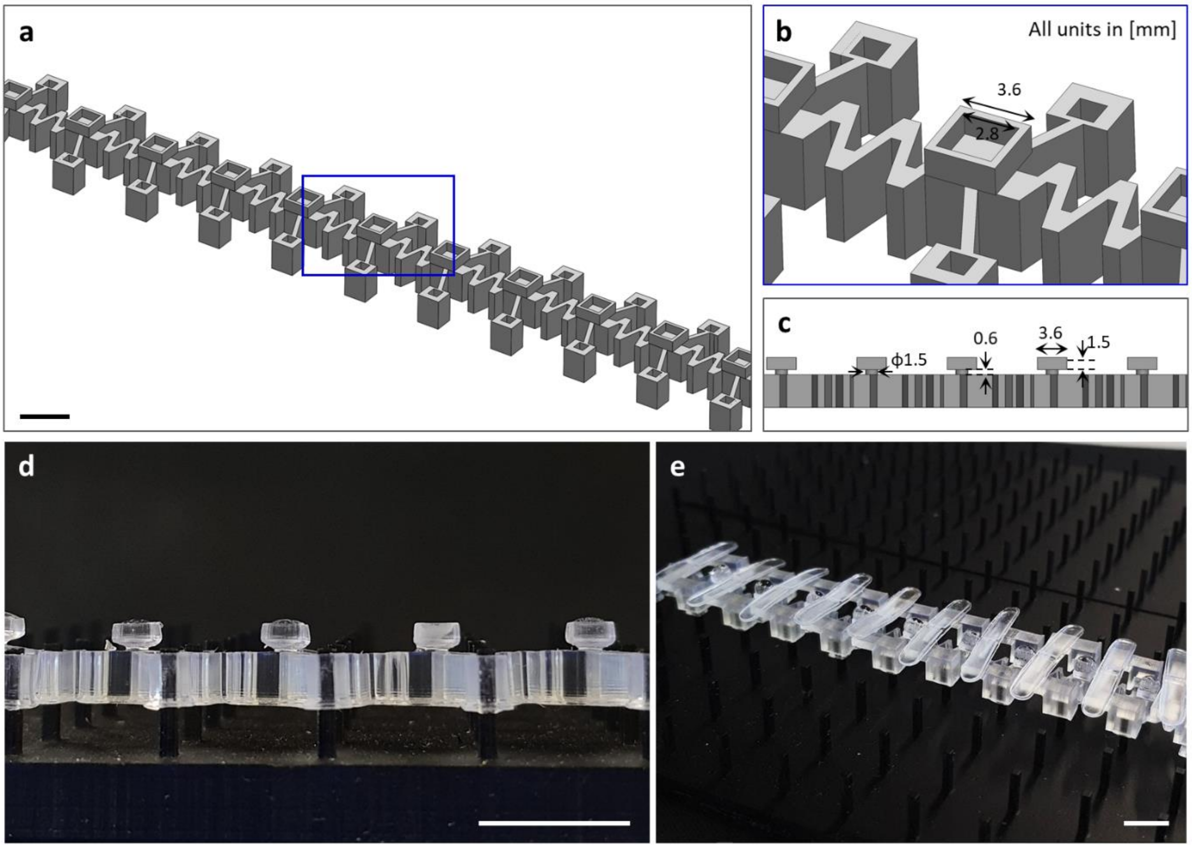

Supplementary Fig. 16 | 3D printed reservoir structures. **a**-**c**, Schematic drawings of the reservoir structure for water droplet transport (**a**), its magnified view (**b**), and side view (**c**). **d**, A side-view image of the 3D printed reservoir structures monolithically mounted onto the mechanical transmission line. **e**, Photograph of the reservoir-integrated transmission line combined with soft hydrogel actuators, reproduced from Fig. 6f. All scale bars, 1 cm.

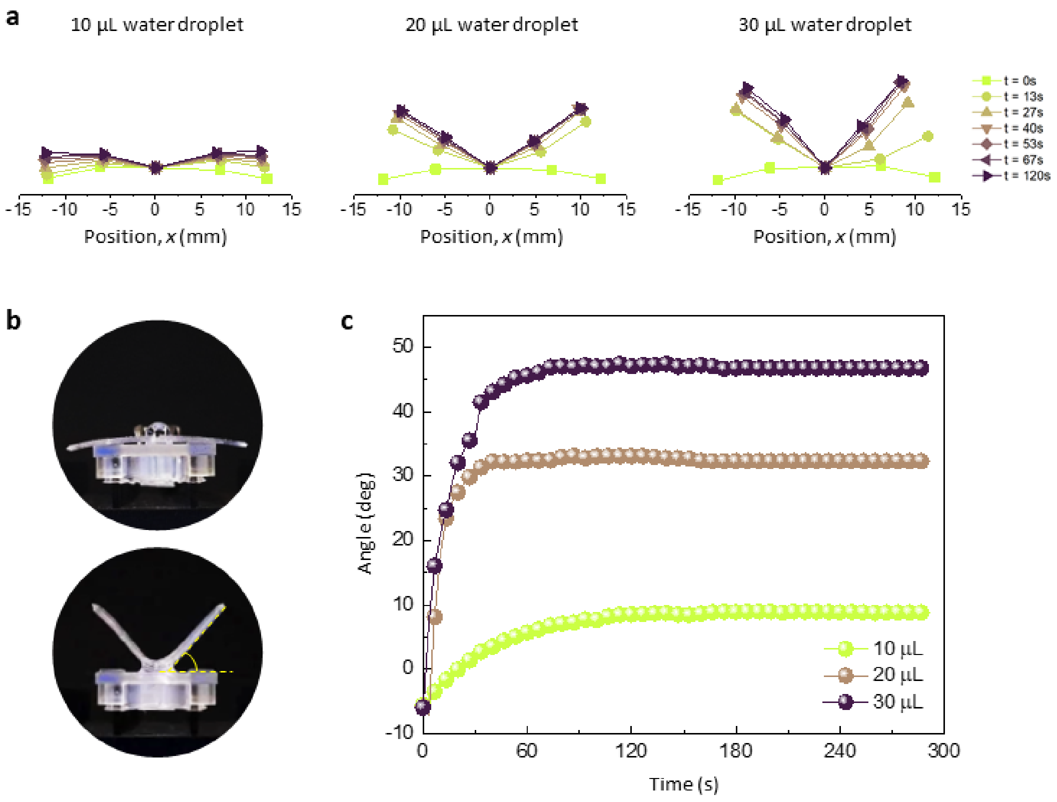

Supplementary Fig. 17 | Experimental characterization of Mimosa-inspired hydrogel actuators. a, Transient responses of the actuators triggered by water droplets with volume of 10, 20, and 30 μL, being transmitted by mechanical solitary waves (Supplementary Movie 10). **b**, Photographs of the soft actuators showing the angle of interest. **c**, Steady-state responses of the soft actuators upon the uptake of different volumes of water.

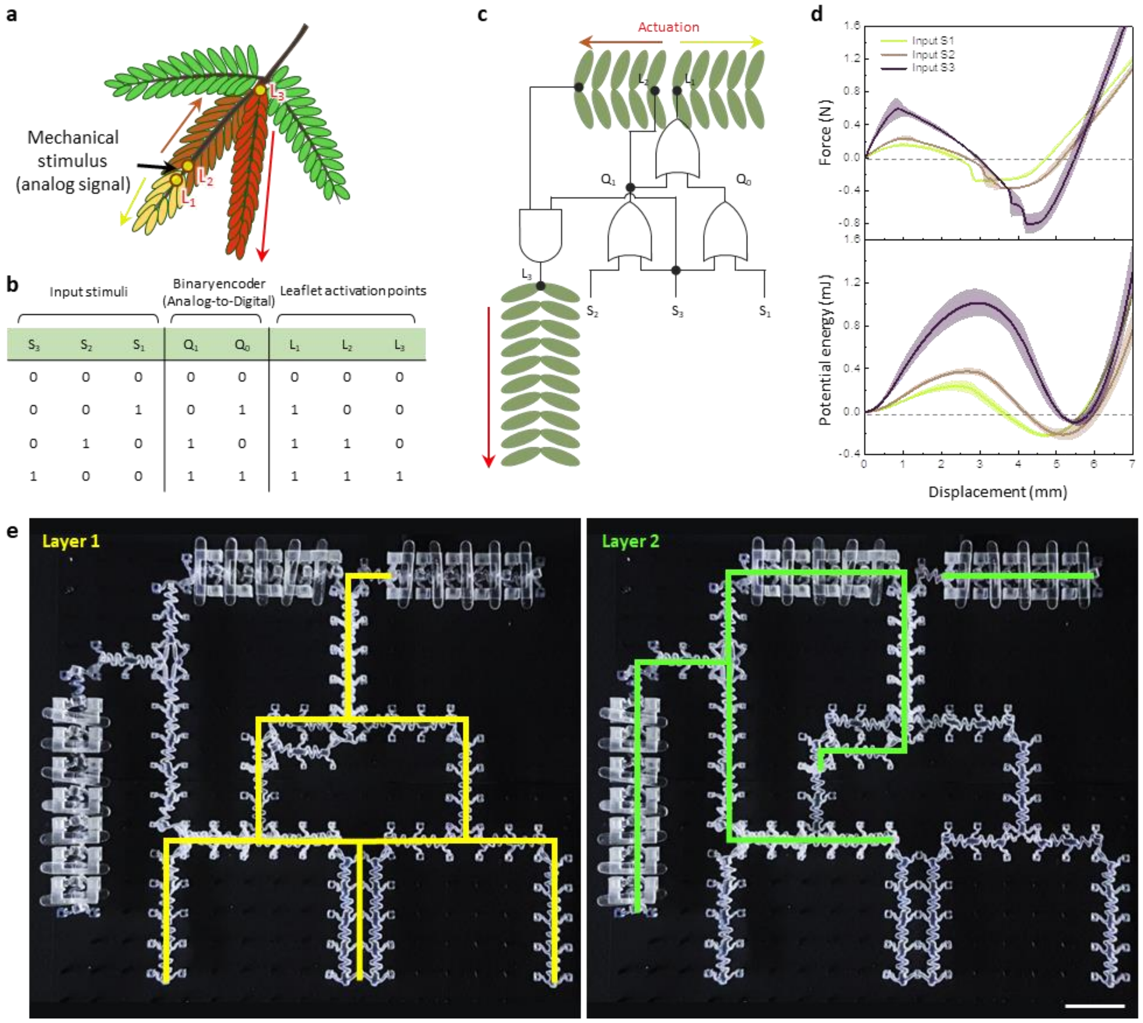

Supplementary Fig. 18 | Design and characterization of Mimosa-inspired autonomous soft machines. a, Schematic illustration of the force-dependent behavior of *M. pudica*. We observe that the propagation pathway of stimuli-induced leaflet folding is regulated by the intensity of the stimulus (Supplementary Movie 9). Given this understanding, we define three representative articulations (L_1, L_2, L_3) that determine the pattern of propagation. **b**, A truth table mapping the force-dependent stimuli (S_1, S_2, S_3) to the decisive activation points (L_1, L_2, L_3). The input signals are converted into 2-bit digital states by a binary encoder (Q_1, Q_0). The outputs are given by: $L_1 = Q_0 + Q_1$, $L_2 = Q_1$, $L_3 = Q_0Q_1$. **c**, Schematic logic diagram of mechanical integrated circuits that implement the truth table in (**b**). **d**, Experimental characterization of input terminals (S_1, S_2, S_3) made of soft bistable beams with different geometric parameters. The error bound indicates SD. **e**, Detailed layout of the double-layered mechanical integrated circuit. Scale bar, 2 cm.

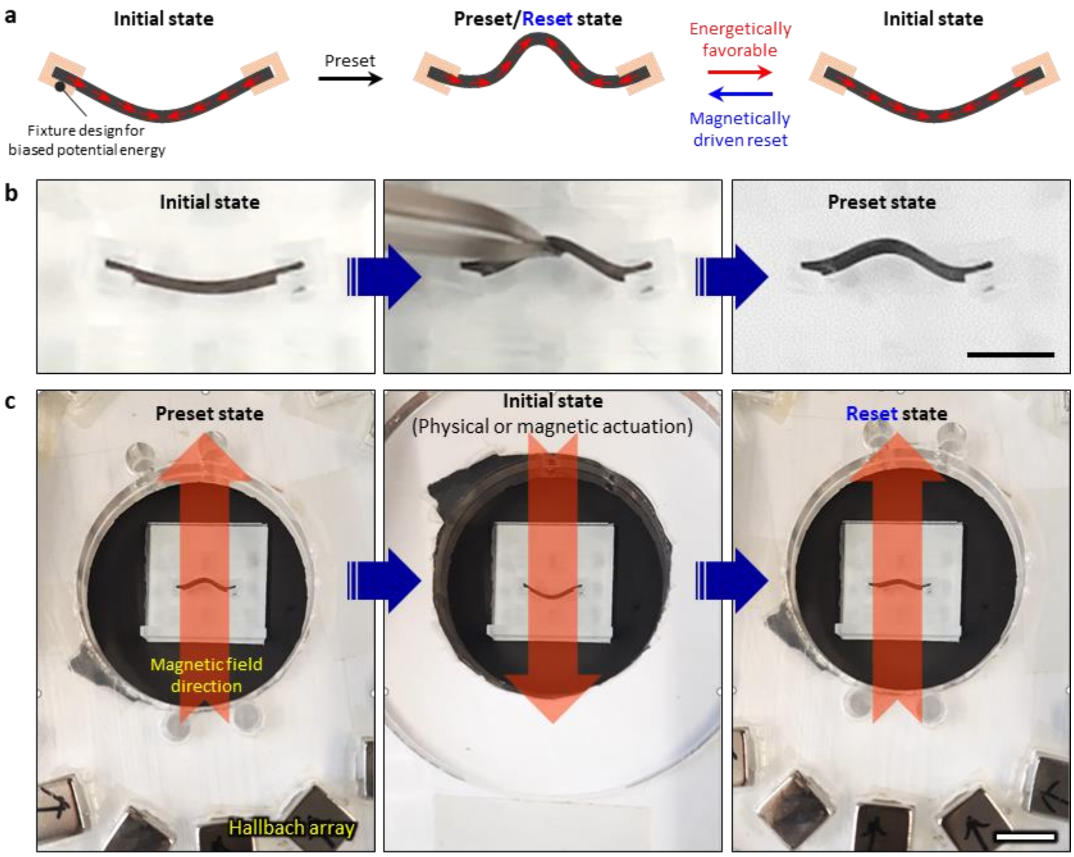

Supplementary Fig. 19 | Preliminary results for magnetically driven resetting. a, Schematics of the unit bistable element consisting of soft magnetic composites with programmed magnetization and its presetting, energetically favorable transition, and magnetically driven resetting processes. **b**, Sequential images of the presetting process in our preliminary experiment. **c**, Sequential images of the magnetically driven resetting process. Scale bars, 5 mm in (**b**), 1 cm in (**c**).
