# Supplementary material for: Integrated mechanical computing for autonomous soft machines
Source: Nat Commun. 2024 Apr 4;15:2933. doi: 10.1038/s41467-024-47201-y (PMC10995184; doi:10.1038/s41467-024-47201-y)
Supplement: Supplementary file 3 — Description of Additional Supplementary Files [file 41467_2024_47201_MOESM3_ESM.pdf]

Description of Additional Supplementary Files

Supplementary Movie 1. Mechanical solitary wave propagation through the mechanical transmission line with and without a computing unit.

Supplementary Movie 2. Simulation of mechanical solitary wave transmission with a mechanical computing unit.

Supplementary Movie 3. NOT gate operation.

Supplementary Movie 4. AND gate operation.

Supplementary Movie 5. OR gate operation.

Supplementary Movie 6. Simulation of 1D cascaded computing.

Supplementary Movie 7. Experimental demonstration of 1D cascaded computing.

Supplementary Movie 8. Experimental demonstration of 2D cascaded computing.

Supplementary Movie 9. Thigmonastic movements of *M. pudica*. Video source: Marco Enrico and WJD, both licensed under CC BY 4.0.

Supplementary Movie 10. A Mimosa-inspired soft hydrogel actuator.

Supplementary Movie 11. A Mimosa-inspired soft machine.

Supplementary Movie 12. A Mimosa-inspired autonomous soft machine based on integrated mechanical computing.
